# A method for sensitivity analysis to assess the effects of measurement error in multiple exposure variables using external validation data

**DOI:** 10.1186/s12874-016-0240-1

**Published:** 2016-10-13

**Authors:** George O. Agogo, Hilko van der Voet, Pieter van ’t Veer, Pietro Ferrari, David C. Muller, Emilio Sánchez-Cantalejo, Christina Bamia, Tonje Braaten, Sven Knüppel, Ingegerd Johansson, Fred A. van Eeuwijk, Hendriek C. Boshuizen

**Affiliations:** 1 Biometris, Wageningen University and Research Centre, Wageningen, The Netherlands; 2 Department of Internal Medicine, Yale University, New Haven, USA; 3 Department of Human Nutrition, Wageningen University and Research Centre, Wageningen, The Netherlands; 4 Nutritional Epidemiology Group, International Agency for Research on Cancer, Lyon, France; 5 Genetic Epidemiology Group, International Agency for Research on Cancer, Lyon, France; 6 Andalusian School of Public Health, Granada, Spain; 7 Department of Hygiene, Epidemiology and Medical Statistics, University of Athens Medical School, Athens, Greece; 8 Department of Community Medicine, University of Tromsø, N-9037 Tromsø, Norway; 9 Department of Epidemiology, German Institute of Human Nutrition Potsdam-Rehbrücke, Nuthetal, Germany; 10 Department of Odontology, Umeå university, Umeå, Sweden; 11 Department of Statistics, mathematical modelling and data logistics, National Institute for Public Health and the Environment (RIVM), Bilthoven, The Netherlands

**Keywords:** Attenuation-contamination matrix, Bayesian MCMC, EPIC study, Measurement error, Validation study

## Abstract

**Background:**

Measurement error in self-reported dietary intakes is known to bias the association between dietary intake and a health outcome of interest such as risk of a disease. The association can be distorted further by mismeasured confounders, leading to invalid results and conclusions. It is, however, difficult to adjust for the bias in the association when there is no internal validation data.

**Methods:**

We proposed a method to adjust for the bias in the diet-disease association (hereafter, association), due to measurement error in dietary intake and a mismeasured confounder, when there is no internal validation data. The method combines prior information on the validity of the self-report instrument with the observed data to adjust for the bias in the association. We compared the proposed method with the method that ignores the confounder effect, and with the method that ignores measurement errors completely. We assessed the sensitivity of the estimates to various magnitudes of measurement error, error correlations and uncertainty in the literature-reported validation data. We applied the methods to fruits and vegetables (FV) intakes, cigarette smoking (confounder) and all-cause mortality data from the European Prospective Investigation into Cancer and Nutrition study.

**Results:**

Using the proposed method resulted in about four times increase in the strength of association between FV intake and mortality. For weakly correlated errors, measurement error in the confounder minimally affected the hazard ratio estimate for FV intake. The effect was more pronounced for strong error correlations.

**Conclusions:**

The proposed method permits sensitivity analysis on measurement error structures and accounts for uncertainties in the reported validity coefficients. The method is useful in assessing the direction and quantifying the magnitude of bias in the association due to measurement errors in the confounders.

**Electronic supplementary material:**

The online version of this article (doi:10.1186/s12874-016-0240-1) contains supplementary material, which is available to authorized users.

## Background

The effect of measurement error on the association between an exposure and an outcome of interest has been studied extensively in epidemiology [1–13], and particularly so in nutritional epidemiology. In nutritional research, the usually weak association between a dietary intake and the risk of a disease can further be distorted by another risk factor that is associated with both the disease and the dietary intake (hereafter, confounder) and by measurement error in the confounder. Moreover, the measurement error in the confounder can be more harmful in distorting the diet-disease association than the measurement error in the dietary intake [6]. If measurement error in the confounder is not taken into account, its effects can resonate so that a dietary intake with no effect can appear to have a sizable effect on the risk of a disease [6]. Resonant confounding due to confounder measurement error can bias the diet-disease association in any direction, even when a researcher adjusts for confounding [6, 14]. The resulting bias can be large [14, 15].

In nutritional research, long-term dietary intakes are generally measured with dietary questionnaires (hereafter, DQs). The DQ is prone to recall bias that can result in either systematic bias or random error [4]. The random error can be due to person-specific bias or within-person variation in intake [16]. To validate the DQ, a validation study is required [17, 18]. In a validation study, a short-term recall instrument or a biomarker is used to obtain unbiased measurements for an intake (hereafter, reference measurements) [18, 19]. The reference measurements are used to quantify the effect of measurement error on the parameter estimate that quantifies the association. The effect of measurement error in the DQ can be quantified with either an attenuation factor or a correlation coefficient between true and measured intake (hereafter, validity coefficient) [4, 16]. The attenuation factor quantifies the bias in the association estimate, whereas the validity coefficient quantifies the loss of statistical power to detect a significant association.

When only one risk factor is measured with error (hereafter, univariate case), a researcher can adjust for the bias in the association by dividing the unadjusted association estimate by the attenuation factor (hereafter, univariate method) [20]. However, complications may arise when confounders are also measured with error (hereafter, multivariate case) [5, 14]. Measurement error in the confounder can contaminate the observed association. In the multivariate case, it is common for both dietary intake and confounder variables to be measured with correlated errors, further influencing the bias. Using the univariate method to adjust for the bias in the multivariate case can lead to substantial bias, especially for strong error correlations [5]. To adjust for the bias in the association using standard methods requires validation data from a validation study [1, 20–22]. Generally, it is very costly to conduct such a validation study in addition to the main study.

We proposed a simple and flexible method to adjust for the bias in the diet-disease association caused by correlated measurement errors, in the absence of internal validation data. The purpose of the proposed method is twofold. First, the method demonstrates how to combine external validation data on the validity of the DQ with the observed DQ data to adjust for the bias in the diet-disease association. Second, the method can be used to conduct sensitivity analysis on the effect of correlated measurement errors on study conclusions.

The method applies a Bayesian method that uses Markov Chain Monte Carlo (MCMC) sampling-based estimation approach [17, 23] and is implemented in SAS version 9.3. We illustrated the proposed method with data from the European Prospective Investigation into Cancer and Nutrition (EPIC) study. The aim in the EPIC example is to adjust for measurement error in self-reported fruits and vegetables intake (hereafter, FV intake), when estimating the association of this dietary exposure with all-cause mortality, while simultaneously adjusting for the self-reported number of cigarettes smoked in a lifetime (hereafter, cigarette smoking), a variable believed to be also associated with all-cause mortality and also measured with error.

## Methods

### The EPIC study example

The EPIC study is an on-going multicentre prospective study to investigate the association between nutrition and chronic diseases such as cancer [24]. In the EPIC cohort, baseline questionnaire and interview data on diet and non-dietary variables, anthropometric measurements and blood samples were collected. The study participants were followed over time for the occurrence of cancer, other diseases and overall mortality. The follow-up questionnaires were used to collect information on selected aspects of lifestyle that are related to the risk of cancer [25]. The EPIC study consisted of about half a million individuals aged mainly between 35 and 70 years, recruited in 23 centres in 10 European countries [24, 26]. Dietary food questionnaires were used to assess long-term dietary intake administered only once per subject. The mortality data were collected at the participating centres through mortality registries or follow-up and death-record collection [25].

We used part of the EPIC data set that consisted of 46758 current smokers who had observed data on self-reported FV intake and self-reported number of cigarettes smoked in a lifetime. Because of the restrictive selection criteria, the selected subset data might not be a representative sample of the entire EPIC cohort; this subset data was used here for illustration and not for inferential purposes. We used FV intake as dietary intake, cigarette smoking as the confounder and whether a person died during the study period as an indicator of all-cause mortality to illustrate the proposed method. We illustrated the method with the aim of adjusting for the bias in the association between FV intake (in 100 g per day) and all-cause mortality, while simultaneously adjusting for confounding by self-reported cigarette smoking and measurement error in cigarette smoking. Note that we did not adjust for other confounding factors.

### A measurement error model for the dietary questionnaire

We consider a Cox proportional hazards model to study the association between FV intake, cigarette smoking and all-cause mortality as

1 $$ \mathrm{H}\left(t\Big|{T}_1,{T}_2\right)={\mathrm{H}}_{\mathrm{o}}\left(\mathrm{t}\right) \exp \left({\beta}_{T_1}{T}_1+{\beta}_{T_2}{T}_2\right), $$

where H_o_(t) is the baseline hazard at time to all-cause mortality $t,\kern0.5em {\beta}_{T_1}$ is the log hazard ratio (hereafter, logHR) for the true long-term FV intake *T*_1_ and ${\beta}_{T_2}$ is the logHR for the true confounder intake (cigarette smoking) *T*_2_. For this study, the main interest is in estimating ${\beta}_{T_1}$. True FV intake, however, is unobservable in practice; therefore, the DQ intake measurement is usually used in place of the unknown true intake. Fitting model (1) to the observed DQ measurements for the FV intake (hereafter, *Q*_1_) and cigarette smoking (hereafter, *Q*_2_), replacing the corresponding true intakes, yields biased logHRs ${\beta}_{Q_1}$ and ${\beta}_{Q_2}$ of ${\beta}_{T_1}$ and ${\beta}_{T_2}$, respectively. We refer to these biased log hazard ratios as unadjusted logHRs. We further denote the vector of unadjusted logHRs ${\left({\beta}_{Q_1},\ {\beta}_{Q_2}\right)}^{\mathrm{T}}$ by *β*_***Q***_ and a vector of true logHRs ${\left({\beta}_{T_1},\ {\beta}_{T_2}\right)}^{\mathrm{T}}$ by *β*_***T***_. We assumed intake reported in the DQ to be linearly related to the true intakes, but with additional measurement errors [4, 16, 27] as

2 $$ {Q}_i={\alpha}_{0i}+{\alpha}_{1i}{T}_i+{\epsilon}_{Q_i},\ i=1,2,\kern0.5em \left(1 = \mathrm{F}\mathrm{V}\ \mathrm{intake},\ 2 = \mathrm{cigarette}\ \mathrm{smoking}\right) $$

where ${\left({\epsilon}_{Q_1},{\epsilon}_{Q_2}\right)}^{\mathrm{T}}={\upepsilon}_{\boldsymbol{Q}}\sim \mathrm{N}\left(\mathbf{0},\ {\Sigma}_{\upepsilon_{\mathbf{Q}}}\right),\kern0.5em {\left({Q}_1,{Q}_2\right)}^{\mathrm{T}}=\boldsymbol{Q};\kern0.5em {\left({\alpha}_{01},{\alpha}_{02}\right)}^{\mathrm{T}}={\boldsymbol{\alpha}}_0,\kern0.5em {\left({\alpha}_{11},{\alpha}_{12}\right)}^{\mathrm{T}}={\boldsymbol{\alpha}}_1;$ the terms in ***α***_**0**_ quantify the constant bias and the terms in ***α***_**1**_ quantify intake-related/proportional scaling bias; the two components ***α***_**0**_ and ***α***_**1**_ jointly quantify systematic bias; the component **ϵ**_***Q***_ is a random error term [16]; ${\epsilon}_{Q_i}$ is assumed to be independent of true intake *T*_*i*_ and the systematic bias components (*α*_0*i*_ and *α*_1*i*_). The random error ${\epsilon}_{Q_i}$ can be split further into two components as ${\epsilon}_{Q_i}={r}_{Q_i}+{\epsilon}_{Q_{e_i}},$ where ${r}_{Q_i}$ is referred to as person-specific bias component that describes the fact that two individuals who consume the same amount of FV or smoke the same number of cigarettes will systematically report their intakes differently; ${\epsilon}_{Q_{e_i}}$ is referred to as the measurement occasion component that is random within an individual. This decomposition of the error term, however, is only possible in the presence of a multiple-replicate study. Noteworthy, it is possible for the magnitude of self-reported intake to depend on the effects of subject’s characteristics such as age and BMI. The contribution of these subject characteristic variables can be incorporated in the measurement error model shown in (2) by adding systematic terms for these subject characteristic variables (for instance, see [28]). Because the interest of this work was not in the effect of subject’s characteristics on the validity of self-report instruments, for simplicity we did not include their effects in the measurement error model. The unadjusted and true logHRs are linked as *β*_***Q***_ = Λ^T^ *β*_***T***_ (for instance, see supplementary information in LS Freedman, A Schatzkin, D Midthune and V Kipnis [21]), where Λ is referred to as attenuation-contamination matrix that quantifies the magnitude of attenuation, including contamination effects (the effects of error in measuring *T*_1_ on ${\beta}_{T_2}$ and the effect of error in measuring *T*_2_ on ${\beta}_{T_1}$) [20, 21]. The diagonal elements of Λ are referred to as attenuation factors and the off-diagonal elements as contamination factors [21].

To adjust for the bias in the association between FV intake and all-cause mortality using the univariate method, a researcher simply divides each unadjusted logHR estimate of FV with the attenuation factor for the FV intake reported on the DQ [21]. Attenuation factor (*λ*) is the ratio of variance of true intake to the variance of measured intake for *i* ^th^ variable, i.e., *λ* = *var*(*T*_*i*_)/*var*(*Q*_*i*_) [7]. Note that this method ignores the contamination effect caused by measurement error in cigarette smoking that is correlated with measurement error in FV intake. In other words, the univariate adjustment method assumes intake measurements for FV intake and cigarette smoking to be uncorrelated. In practice, however, these variables are expected to be correlated through their true intakes, measurement errors or through both components.

To adjust for the bias in the association between FV intake and all-cause mortality using the multivariate method that accounts for correlation of measured FV intake and measured cigarette smoking, a researcher applies the inverse of the attenuation-contamination matrix to the unadjusted logHRs as [20, 21]

3 $$ {\widehat{\beta}}_{\boldsymbol{T}}={\left({\widehat{\varLambda}}^{\mathrm{T}}\right)}^{-1}{\widehat{\beta}}_{\boldsymbol{Q}}, $$

where $\widehat{\varLambda}$ is usually estimated from a validation study. Noteworthy, expression (3) is simply an extension of the univariate formula to a multidimensional setting with more than one variable measured with error. Many epidemiologic studies, however, do not include validation studies besides the main study, because validation studies are costly. We, therefore, propose a method that incorporates external information on the validity of self-report instruments in estimating *Λ*. If *Q*_*i*_ is assumed to be measured with no systematic bias (i.e., *α*_0*i*_ = 0, *α*_1*i*_ = 1 for both FV intake and cigarette smoking), *Λ* is the product of two covariance matrices: Σ_**T**_ for true intakes and Σ_**Q**_^− 1^ for the inverse of the covariance matrix of self-report intakes in the DQ and is estimated as $\widehat{\varLambda}={\widehat{\sum}}_{\mathbf{T}}{{\widehat{\sum}}_{\mathbf{Q}}}^{-1}$ (see RJ Carroll, D Ruppert, LA Stefanski and CM Crainiceanu [1], p.362). Without systematic bias the elements required to obtain $\widehat{\varLambda}$ are:

4 $$ \widehat{\varLambda}=\left(\begin{array}{cc}\hfill {\widehat{\sigma}}_{T_1}^2\hfill & \hfill {\widehat{\sigma}}_{T_1{T}_2}\hfill \\ {}\hfill {\widehat{\sigma}}_{T_1{T}_2}\hfill & \hfill {\widehat{\sigma}}_{T_2}^2\hfill \end{array}\right){\left(\begin{array}{cc}\hfill {\widehat{\sigma}}_{Q_1}^2\hfill & \hfill {\widehat{\sigma}}_{Q_1{Q}_2}\hfill \\ {}\hfill {\widehat{\sigma}}_{Q_1{Q}_2}\hfill & \hfill {\widehat{\sigma}}_{Q_2}^2\hfill \end{array}\right)}^{-1}, $$

where ${\widehat{\sigma}}_{T_1}^2$ and ${\widehat{\sigma}}_{T2}^2$ are variance estimates of *T*_1_ and *T*_2_, respectively. Since ${\widehat{\sum}}_{\mathbf{Q}}$ can be estimated directly from the observed DQ data, the task is to obtain ${\widehat{\sigma}}_{T_1}^2,{\widehat{\sigma}}_{T_2}^2$ and ${\widehat{\sigma}}_{T_1{T}_2}$ in order to estimate all the elements in Λ shown in expression (4).

The covariance between true intakes is ${\widehat{\sigma}}_{T_1{T}_2}={\widehat{\rho}}_{T_1{T}_2}{\widehat{\sigma}}_{T_1}{\widehat{\sigma}}_{T_2}$ and the covariance between the observed intakes reported in the DQ is

5 $$ {\widehat{\sigma}}_{Q_1{Q}_2}={\widehat{\rho}}_{T_1{T}_2}{\widehat{\sigma}}_{T_1}{\widehat{\sigma}}_{T_2}+{\widehat{\rho}}_{\epsilon_{Q_1}{\epsilon}_{Q_2}}{\widehat{\sigma}}_{\epsilon_{Q_1}}{\widehat{\sigma}}_{\epsilon_{Q_2}}, $$

where ${\widehat{\rho}}_{T_1{T}_2}$ is the estimate of correlation between true intakes and ${\widehat{\rho}}_{\epsilon_{Q_1}{\epsilon}_{Q_2}}$ is the estimate of correlation between the errors.

### Estimation of **Σ**_**T**_ from DQ measurements and external validation data

We used the validity coefficients for the DQ to estimate the variance components of true intakes ${\sigma}_{T_1}^2$ and ${\sigma}_{T_2}^2$. Using parameters in the model shown in expression (2), the validity coefficient for the DQ is given by [4, 16]

$$ {\rho}_{Q_i{T}_i}=\frac{cov\left({Q}_i,{T}_i\right)}{\sqrt{var\left({Q}_i\right)var\left({T}_i\right)}}=\frac{\alpha_{1i}\ {\sigma}_{T_i}}{\sigma_{Q_i}}\kern0.5em \left(i=1,2\right). $$

From the validity coefficient formula, the variance for the true intake ${\sigma}_{T_i}^2$ can be estimated as

6 $$ {\widehat{\sigma}}_{T_i}^2 = {\left(\frac{{\widehat{\rho}}_{Q_i{T}_i}}{{\widehat{a}}_{1i}}\ {\widehat{\sigma}}_{Q_i}\right)}^2,\ i=1,\ 2\kern0.5em \left(1 = \mathrm{F}\mathrm{V}\ \mathrm{intake},\ 2 = \mathrm{cigarette}\ \mathrm{smoking}\right). $$

Thus, to obtain ${\widehat{\sigma}}_{T_i}^2$, we need external validation data on the validity coefficient ${\rho}_{Q_i{T}_i}$ and the proportional scaling bias term *α*_1*i*_. Hereafter, we set the proportional scaling bias term to one (*α*_1*i*_ = 1). The reason is that, at the time of this work, there were no previous studies with information on *α*_1*i*_ for FV intake and number of cigarettes smoked in a lifetime. However, this term can be incorporated in the measurement error model when dealing with study variables where information on systematic bias components is available, including this bias also in formula 4.

To obtain ${\widehat{\sigma}}_{T_1{T}_2}$ one has to make assumptions, as this information is generally not available from studies. The assumption can either be made directly on the correlation between true intakes ${\widehat{\rho}}_{T_1{T}_2}$ or indirectly on the correlation between the errors ${\widehat{\rho}}_{\epsilon_{Q_1}{\epsilon}_{Q_2}}$ using expression (5). The choice depends on the available prior knowledge for the study variables. The advantage of the proposed method is that it permits the user to make the assumption on either of the two correlations. A general assumption is that individuals who consume dietary intakes with health benefits will often systematically over report their intakes, leading to positively correlated errors between variables with health benefits. Also, these same individuals will often tend to systematically under report intakes with harmful effects, leading to positively correlated errors between these variables with harmful effects. Conversely, if the same individuals who systematically over report their dietary intakes with health benefits also systematically under report their intakes with harmful effects, then one would expect negatively correlated errors between these reported intakes. We obtained a plausible range of validity coefficients from a literature review of studies on the validity of the questionnaire as a self-report instrument for long-term dietary intake *T*_1_ and confounder intake *T*_2_. We equated the minimum and maximum validity coefficients ${\rho}_{Q_i{T}_i}$ obtained from the literature to plausible quantiles of the uncertainty distribution. As no data are available for either of the two correlation coefficients ${\rho}_{\epsilon_{Q_1}{\epsilon}_{Q_2}}$ or ${\rho}_{T_1{T}_2}$ for these two study variables, we assumed a range of possible values for these correlation coefficients, thus accounting for uncertainty due to heterogeneity between study populations in the literature reports.

### A description of the proposed multivariate measurement error adjustment method

To adjust for the bias in the association parameters, we propose a method that combines the observed self-report data in the DQ with the external validity information for the DQ derived from the literature. The method uses a Bayesian approach and MCMC estimation technique. This method accounts for the uncertainty in the literature reports, uncertainty that is both due to heterogeneity in the study populations in the literature reports and in the parameter estimation. Here, we describe the bias-adjustment steps for the proposed method.

First, we obtained the posterior distributions of the unadjusted logHR estimates ${\left({\widehat{\beta}}_{Q_1},\ {\widehat{\beta}}_{Q_2}\right)}^{\mathrm{T}}$. This was done by fitting a Bayesian Cox proportional hazards model shown in (1) to the observed self-report data in the DQ for FV intake and cigarette smoking. In the Bayesian Cox model, we assumed weakly informative independent normal priors ${\pi}_{\beta_{Q_i}}$ for the unadjusted logHRs by choosing a large variance as ${\pi}_{\beta_{Q_i}}\sim \mathrm{N}\left(0,\ {10}^6\right)$.

Second, we estimated the posterior distribution of the covariance matrix for the observed self-report DQ data ( Σ_**Q**_). Based on exploration of the DQ data, a normal distribution was assumed for the self-report intake data as **Q** ~ N(μ_**Q**_, Σ_**Q**_). To ensure minimal influence of the prior information on the estimate of Σ_**Q**_, a weakly informative inverse Wishart prior $\left({\pi}_{\Sigma_{\mathbf{Q}}}\right)$ was assumed as ${\pi}_{\Sigma_{\mathbf{Q}}}\sim \mathrm{I}\mathrm{W}\left({\Lambda}_0,\ {\upupsilon}_0\right)$, where Λ_0_ = **I**_2_ (identity matrix) is the scale parameter and *υ*_0_ = 2 is the degrees of freedom. Note, this parameterization ensures a weakly informative inverse Wishart prior for Σ_**Q**_ [23]. Noteworthy, varying the magnitude of *υ*_0_ did not alter the results much, because the likelihood dominated the prior, given the large size of the EPIC data set.

Third, we generated the validity coefficients for FV intake and cigarette smoking using prior information from the literature on external validation studies. We interpreted the lower and upper limits for the literature-reported validity coefficients as 0.05 and 0.95 quantiles of the distribution of plausible values, respectively. The validity coefficients were generated in a Fisher-z transformed scale as explained in Additional file 1: Appendix A. The generated validity coefficients were transformed back to the original scale using the inverse of Fisher-z transformation.

Fourth, using the validity coefficients generated from the literature data $\left({\rho}_{Q_i{T}_i}\right)$ and the posterior distribution for the variances of self-report intakes $\left({\sigma}_{Q_i}^2\right)$ estimated from the observed DQ data for FV intake and cigarette smoking, the corresponding distribution for the variance of true intakes $\left({\sigma}_{T_i}^2\right)$ was estimated as ${\sigma}_{T_i}^2={\left({\widehat{\rho}}_{Q_i{T}_i} \times {\widehat{\sigma}}_{Q_i}\right)}^2$ using expression (6), but with *α*_1*i*_ set to one.

Lastly, in order to estimate all the elements of Λ, we needed to estimate the covariance between true intakes ${\widehat{\sigma}}_{T_1{T}_2}$. This could be done by decomposing the covariance in the observed DQ data ${\widehat{\sigma}}_{Q_1{Q}_2}$ into the unknown covariance between true intakes ${\widehat{\sigma}}_{T_1{T}_2}$ and the unknown covariance between the errors ${\widehat{\sigma}}_{\epsilon_{Q_1}{\epsilon}_{Q_2}}$, when *α*_1*i*_ is set to one as shown in expression (5). This covariance decomposition is only possible by making plausible prior assumption on either of the two covariances. Here, we made an assumption on the plausible range of the correlation between the errors, because making this assumption is more intuitive for the two study variables in this work. To estimate the covariance between the errors ${\widehat{\sigma}}_{\epsilon_{Q_1}{\epsilon}_{Q_2}}$, the error variance ${\widehat{\sigma}}_{\epsilon_{Q_i}}^2$ was calculated as the difference between the estimated variance in the observed DQ data ${\widehat{\sigma}}_{Q_i}^2$ and the estimated variance in true intake data ${\widehat{\sigma}}_{T_i}^2$ as ${\widehat{\sigma}}_{\epsilon_{Q_i}}^2={\widehat{\sigma}}_{Q_i}^2\left(1-{\rho}_{Q_i{T}_i}^2\right)$. The remaining task is to estimate the unknown correlation between the errors $\left({\widehat{\rho}}_{\epsilon_{Q_1}{\epsilon}_{Q_2}}\right)$ required to obtain ${\widehat{\sigma}}_{\epsilon_{Q_1}{\epsilon}_{Q_2}}$. To our knowledge, there were no previous studies at the time of this work with information on the error correlation between FV intake and the number of cigarettes smoked in a lifetime. Due to lack literature data on this error correlation, we generated the correlation between the errors ${\rho}_{\epsilon_{Q_1}{\epsilon}_{Q_2}}$ from a plausible range, guided by the correlation in the observed DQ data and the prior information on the most probable sign of the correlation between the errors in the FV intake and cigarette smoking (as explained in the next section). With the generated ${\rho}_{\epsilon_{Q_1}{\epsilon}_{Q_2}}$, we could therefore obtain ${\widehat{\sigma}}_{T_1{T}_2}$ as the difference between ${\widehat{\sigma}}_{Q_1{Q}_2}$ and ${\widehat{\sigma}}_{\epsilon_{Q_1}{\epsilon}_{Q_2}}$ parametrized as ${\widehat{\sigma}}_{T_1{T}_2} = {\widehat{\sigma}}_{Q_1{Q}_2}-{\rho}_{\epsilon_{Q_1}{\epsilon}_{Q_2}}{\widehat{\sigma}}_{Q_1}{\widehat{\sigma}}_{Q_2}\ \sqrt{\left(1-{\rho}_{Q_1{T}_1}^2\right)\left(1-{\rho}_{Q_2{T}_2}^2\right)}$. Thus, the distribution of the adjusted logHR for FV intake $\left({\widehat{\beta}}_{T_1}\right)$ could be estimated from the joint distribution of ${\left({\widehat{\varLambda}}^{\mathrm{T}}\right)}^{-1}{\widehat{\beta}}_{\boldsymbol{Q}}$ as shown in expression (3) and by following the above steps.

### A comparison of the proposed method with the univariate method

We compared the results from the proposed multivariate method with (i) the results from applying the univariate method that ignores confounding by cigarette smoking and (ii) with the results from a method that ignores measurement error.

The proposed method was implemented in SAS version 9.3 using the MCMC procedure as follows. The distributions of Fisher z-transformed validity coefficients were sampled directly from their prior distributions as explained above. The posterior distributions for the unadjusted logHRs estimates in the Bayesian Cox proportional hazard model were sampled using the N-Metropolis method, with all initial parameter values set to zero. The convergence of the chains was assessed with trace plots and autocorrelation with autocorrelation plots. The analysis was based on 50 000 posterior samples, after discarding 5000 burn-in samples and using 5000 samples to tune the parameters (Additional file 1: Appendix C). The results were summarized with density plots and posterior summary measures. We used R version 2.15.2 for graphing.

### Sensitivity analysis

In our example, we investigated how different assumptions on the extent of measurement error in cigarette smoking affected the estimated logHR of FV intake ${\widehat{\beta}}_{T_1}.$ To do this, we used different values for the validity coefficients that were within the range reported in the literature. For each selected value of the validity coefficient, ${\beta}_{T_1}$ was estimated using the proposed adjustment method and then compared with the unadjusted estimate. We further assessed how ${\widehat{\beta}}_{T_1}$ varied with the magnitude of the correlation between the errors in FV intake and cigarette smoking. This helps to assess the sensitivity of the estimates to different magnitudes of the correlation between the errors. Lastly, we investigated the sensitivity of the results to the level of the uncertainty (expressed in quantile interval) assigned to the limits of the validity coefficients reported from the literature.

### External data for FV intake and cigarette smoking

According to a pilot study on evaluation of dietary intake measurements in the EPIC study in nine European countries by R Kaaks, N Slimani and E Riboli [29] and a review of validation studies on measuring FV intake in EPIC study and in similar populations by A Agudo [30], the validity coefficients of the DQ in measuring long-term FV intake is usually reported between 0.3 and 0.7. This range is consistent with the results reported from other similar validation studies [31–33]. A validity coefficient greater or equal to 0.9 was considered as very uncommon [30].

According to Stram, Huberman and Wu [34], the validity coefficient of self-reported number of cigarettes smoked ranges mostly from 0.4 to 0.7. This range is consistent with the findings from other similar validation studies on adult smokers [35–37]. In particular, in a study on validation of self-reported smoking for 36 volunteers aged between 20 and 36 years by Eliopoulos [36], the correlation between the number of cigarettes smoked per day and nicotine levels in the hair and plasma was reported between 0.48 and 0.63. With cotinine levels in the hair and plasma, this correlation was reported between 0.57 and 0.63. In the same study, a good correlation of 0.70 was observed between self-reported number of cigarettes smoked and carboxyhaemoglobin. A validity coefficient greater or equal to 0.85 was considered as very high [34]. We interpreted these reported lower and upper limits of the validity coefficients as the 0.05 and 0.95 quantiles of the uncertainty distribution, respectively. The chosen limit of the uncertainty distribution allows for all plausible values outside the reported range and accounts for the population heterogeneity in these literature studies (see Additional file 1: Appendix B).

Particular to FV intake and cigarette smoking, we assumed the error correlation to be mostly negative, because an individual who tends to systematically over report his FV intake (a healthy habit) will likely under report his cigarette smoking (an unhealthy habit). The assumed magnitude of error correlation, however, must be compatible with the correlation in the observed data such that the covariance in the observed data should equal the sum of the assumed covariance between true intakes and the assumed covariance between the errors. To ensure this compatibility, we obtained the upper limit of error correlation in the case that the correlation between true intakes is zero (i.e., the error covariance equals the covariance in the observed data) and assumed zero as the lower limit (i.e., the covariance in the observed data equals the covariance between true intakes).

## Results

Table 1 describes the logHR estimate for FV intake (per 100g per day) and average number of cigarettes smoked per day, adjusted for the bias with the multivariate and the univariate methods; also shown are the unadjusted estimates. The adjusted estimates presented in this Table were obtained by using the following 90 % CI represented by (lower-upper) limits for the validity coefficients in estimating the variances for true intakes: 0.3–0.7 for FV intake, and 0.4–0.7 for cigarette smoking; the distribution of error correlation was estimated as explained above. The logHR estimate adjusted for the bias with either the multivariate or the univariate method is greater in absolute value than the unadjusted estimate. The estimate adjusted for the bias with the multivariate method shows an about fourfold increase in the strength of association as compared with the unadjusted estimate. A similar magnitude of adjustment is shown with the univariate method. For cigarette smoking, both bias-adjustment methods give similar values for the logHR estimate. Further, the logHR for FV intake is estimated with a slightly larger uncertainty than the logHR for cigarette smoking. The similarity in the performance of the two bias-adjustment methods is due to the weak negative correlation between the errors that is compatible with the correlation in the observed data (here, ${\widehat{\rho}}_{Q_1{Q}_2}=-0.07$). The weak error correlation leads to a minimal contamination effect due to confounding by cigarette smoking. As expected, the variability in the unadjusted estimate is much smaller than the variability in the adjusted estimates for both intake variables. The small variability observed in the unadjusted estimates is because there is no uncertainty involved when measurement error is ignored in estimating the log hazard ratios.

**Table 1:** The mean (standard deviation), median, 0.05 and 0.95 quantiles, and mode for the Log Hazard Ratio (logHR) estimates for FV intake (per 100g per day) and average number of cigarettes smoked (per day) adjusted for the bias with multivariate and univariate methods, and also the unadjusted estimates that ignore measurement error, EPIC study 1992–2000

	LogHR estimate for FV intake ${\widehat{\beta}}_{T_1}$				LogHR estimate for cigarettes smoking ${\widehat{\beta}}_{T_2}$			
Methods^a^	mean (SD)	median	90 % CI	mode	mean (SD)	median	90 % CI	mode
Multivariate	-0.181 (0.090)	-0.157	-0.375, -0.078	-0.125	0.163 (0.079)	0.145	0.094;0.294	0.125
Univariate	-0.169 (0.082)	-0.147	-0.339, -0.077	-0.117	0.162 (0.077)	0.143	0.093;0.290	0.123
Unadjusted	-0.042 (0.007)	-0.042	-0.053, -0.031	-0.042	0.046 (0.002)	0.046	0.043;0.049	0.046

*Abbreviation*: CI is level of uncertainty in the range of literature-reported validity coefficient ${\rho}_{T_i{Q}_i}$ expressed as a credible interval

^a^The results shown above were obtained by using the following (lower-upper) limits for the validity coefficients in estimating the variances for true intakes: 0.3–0.7 for FV intake, and 0.4–0.7 for cigarette smoking

**Fig. 1:**
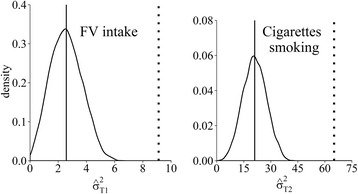
Kernel densities for the estimated posterior samples of variances for true intake levels of fruit and vegetable intake (FV intake, left panel) and true number of cigarettes smoked (right panel). The dotted vertical lines show the variance estimates from self-report in the DQ and the solid vertical lines show the posterior means of the estimated variances for true intake distributions

Figure 1 displays the distribution for the estimates of the variance components required to estimate the attenuation-contamination matrix. The figure presents the kernel densities (curves) and means (solid vertical lines) of the variance estimates of the true intake levels and the mean estimate for the variance from the DQ measurements (dotted vertical lines) for FV intake (left panel) and cigarette smoking (right panel). From the graph, a large percentage of variability in the DQ is seemingly due to measurement error, and is influenced by the assumed distribution for the validity coefficient. Based on this assumption, about 70 % of variability in the DQ for both variables is due to measurement error. This means that only about 30 % of the variability is attributable to inter-individual variability in true intake. The width of the density plot portrays the level of uncertainty involved in estimating the variance of true intake.

**Fig. 2:**
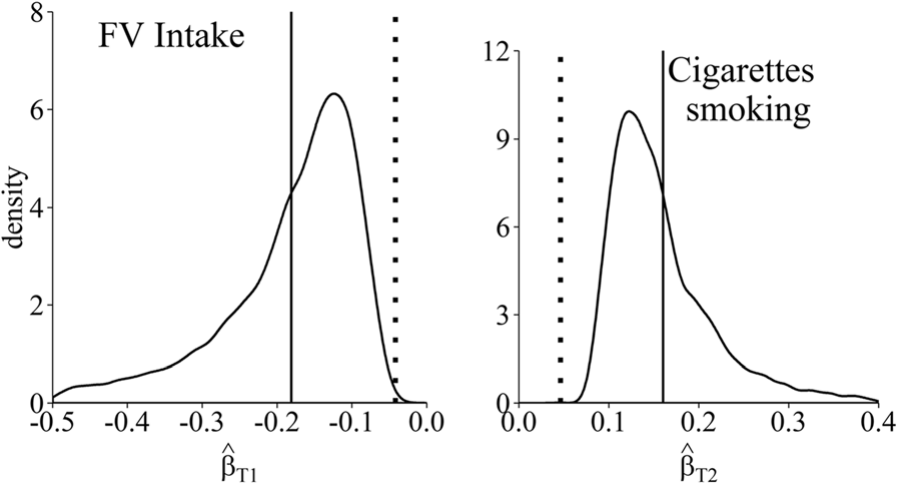
The kernel densities for the distribution of logHR estimates for fruits and vegetable intake per 100 g per day (${\widehat{\beta}}_{T1}$, left panel) and for the number of cigarettes smoked per day (${\widehat{\beta}}_{T2}$, right panel) adjusted for the bias with the multivariate method. The dotted vertical line indicates the means of unadjusted logHR estimates; the solid vertical lines indicate the means of logHR estimates adjusted for the bias

Figure 2 shows the kernel densities and the means (solid vertical lines) for the estimates obtained with the multivariate method using the same limits for the validity coefficients and the estimation method for the error distribution as explained earlier. The dotted vertical lines show the means of the unadjusted estimates. On average, the adjusted estimates are greater in absolute values than the unadjusted estimates, suggesting a stronger beneficial effect of FV intake (left panel) and stronger harmful effect of cigarette smoking (right panel). Importantly, in the multivariate case when both variables are measured with correlated errors, the unadjusted estimates can sometimes underestimate or overestimate the association, as hinted by the part of the distribution where ${\widehat{\beta}}_{T_1}<{\overline{\widehat{\beta}}}_{Q_1}$ (left panel). The method estimates ${\beta}_{T_1}$ with larger uncertainty (wider width) than ${\beta}_{T_2}$.

Presented further are the results from the sensitivity analyses. Table 2 presents the mean (standard deviation), median and mode of the logHR estimate for FV intake ${\widehat{\beta}}_{T_1}$ and cigarette smoking ${\widehat{\beta}}_{T_2}$ for various magnitudes of the validity coefficients of self-reported FV intake ${\rho}_{T_1{Q}_1}$ and self-reported cigarette smoking ${\rho}_{Q_2{T}_2}$. It is evident that the logHR estimate for FV intake ${\widehat{\beta}}_{T_1}$ is influenced by the extent of measurement error assumed for cigarette smoking. For instance, when the validity coefficient for FV intake $\left({\rho}_{Q_1{T}_1}\right)$ is assumed as 0.5 and the validity coefficient for cigarette smoking $\left({\rho}_{Q_2{T}_2}\right)$ varies from 0.5 to 0.7, ${\widehat{\beta}}_{T_1}$ is altered by about -3.8 % (from -0.182 to -0.175). In contrast, the assumed magnitude of error in FV intake does not importantly influence the logHR estimate for the effect of cigarette smoking $\left({\widehat{\beta}}_{T_2}\right)$; for instance, when ${\rho}_{Q_2{T}_2}$ is assumed as 0.5 and ${\rho}_{Q_1{T}_1}$ varies from 0.5 to 0.7, the value of ${\widehat{\beta}}_{T_2}$ is almost the same. Noteworthy, if substantial measurement error is assumed for cigarette smoking, ${\widehat{\beta}}_{T_1}$ can become smaller than the unadjusted estimate, even when FV intake is assumed to be measured without error. The precision of the logHR estimates declines when larger measurement error is assumed for both variables. As expected, when both variables are assumed to be measured without error $\left({\rho}_{T_1{Q}_1}={\rho}_{T_2{Q}_2}=1\right)$ we get the same results as the unadjusted estimates.

**Table 2:** The mean (standard deviation), median and mode of log hazard ratio estimates for fruit and vegetables (FV) intake and number of cigarettes smoked adjusted for the bias with the multivariate method in the sensitivity analysis by varying magnitudes of validity coefficients assumed for the DQs for FV intake $\left({\rho}_{T_1{Q}_1}\right)$ and cigarettes $\left({\rho}_{T_2{Q}_2}\right)$, EPIC study 1992–2000

Validity coefficient^a^		LogHR estimate for FV intake ${\widehat{\beta}}_{T_1}$			LogHR for cigarettes smoking ${\widehat{\beta}}_{T_2}$		
${\rho}_{T_1{Q}_1}$	${\rho}_{T_2{Q}_2}$	mean (SD)	median	mode	mean (SD)	median	mode
0.3	0.3	-0.622 (0.227)	-0.605	-0.567	0.546 (0.048)	0.537	0.527
	0.5	-0.520 (0.101)	-0.517	-0.508	0.191 (0.012)	0.190	0.189
	0.7	-0.493 (0.083)	-0.491	-0.484	0.096 (0.005)	0.096	0.096
0.5	0.3	-0.207 (0.067)	-0.206	-0.203	0.522 (0.024)	0.522	0.521
	0.5	-0.182 (0.033)	-0.182	-0.181	0.187 (0.008)	0.187	0.187
	0.7	-0.175 (0.028)	-0.174	-0.173	0.095 (0.004)	0.095	0.095
0.7	0.3	-0.098 (0.030)	-0.097	-0.096	0.517 (0.021)	0.517	0.518
	0.5	-0.090 (0.017)	-0.090	-0.090	0.186 (0.008)	0.186	0.186
	0.7	-0.088 (0.015)	-0.088	-0.088	0.095 (0.004)	0.095	0.095
1.0	0.3	-0.029 (0.009)	-0.029	-0.029	0.513 (0.021)	0.514	0.517
	0.5	-0.038 (0.007)	-0.038	-0.038	0.185 (0.008)	0.185	0.185
	0.7	-0.041 (0.007)	-0.040	-0.040	0.094 (0.004)	0.094	0.095
	1.0	-0.042 (0.007)	-0.042	-0.042	0.046 (0.002)	0.046	0.046

^a^ The validity coefficients used here for the sensitivity analysis are within the range reported in the literature

Presented in Table 3 are the summary results for the logHR estimates adjusted for the bias with the proposed multivariate method by varying the assumed error correlation from -0.2 to 0.10 in the sensitivity analysis. It is evident that the magnitude of error correlation affects the mean estimate of the logHR for FV intake more than the mean estimate of the logHR for cigarette smoking. For positively correlated errors, though not expected for the two study variables, the mean adjusted logHR estimate $\left({\widehat{\beta}}_{T_1}=-0.32\right)$ even becomes smaller in absolute value than the unadjusted estimate. Further, we compare the results obtained by assuming uncorrelated errors $\left({\rho}_{\epsilon_1{\epsilon}_2}=0\right)$ in Table 3 with the results in Table 1. From this comparison, it is evident that the difference between the estimates obtained with the multivariate and univariate methods is due to the assumed magnitude of the correlation between true intakes $\left({\rho}_{T_1{T}_2}\right)$. When the errors are assumed to be uncorrelated, the presence of ${\rho}_{T_1{T}_2}$alters${\widehat{\beta}}_{T_1}$ by about -6 %, i.e., from -0.169 to -0.159 as estimated with the univariate method and the multivariate method, respectively.

**Table 3:** The mean (standard deviation), median, 0.05 and 0.95 quantiles and mode of the log hazard ratio estimates adjusted for the bias with the multivariate method in the sensitivity analysis by varying the magnitude of error correlation between DQ measurements for FV intake and average number of cigarettes smoked in a lifetime, EPIC study 1992–2000

Correlations		LogHR estimate for FV intake ${\widehat{\beta}}_{T_1}$				LogHR for cigarettes smoking ${\widehat{\beta}}_{T_2}$			
${\rho}_{\epsilon_1{\epsilon}_2}$	${\overline{\widehat{\rho}}}_{T_1{T}_2}$	mean (SD)	median	90 % CI	mode	mean (SD)	median	90 % CI	mode
-0.20	0.51	-0.301 (0.098)	-0.294	-0.471, -0.155	-0.237	0.183 (0.064)	0.169	0.109, 0.304	0.151
-0.15	0.38	-0.277 (0.099)	-0.264	-0.460, -0.137	-0.212	0.178 (0.067)	0.163	0.105, 0.305	0.143
-0.10	0.24	-0.247 (0.098)	-0.228	-0.440, -0.117	-0.178	0.173 (0.070)	0.156	0.101, 0.303	0.135
-0.05	0.10	-0.207 (0.093)	-0.184	-0.403, -0.096	-0.143	0.167 (0.075)	0.148	0.097, 0.295	0.130
0.00	-0.04	-0.159 (0.083)	-0.136	-0.337, -0.069	-0.106	0.161 (0.083)	0.141	0.093, 0.286	0.118
0.10	-0.32	-0.038 (0.098)	-0.045	-0.171, 0.126	-0.047	0.157 (0.075)	0.137	0.087, 0.294	0.116

*Abbreviation*: CI is level of uncertainty in the range of literature-reported validity coefficient ${\rho}_{T_i{Q}_i}$ expressed as a credible interval;

${\overline{\widehat{\rho}}}_{T_1{T}_2}$ is posterior mean estimate for the correlation coefficient between true intake variables

Table 4 presents the mean (standard deviation), median, 0.05 and 0.95 quantiles and mode for logHR estimates ${\widehat{\beta}}_{T_1}$ and ${\widehat{\beta}}_{T_2}$ adjusted for the bias with the proposed multivariate method for various possibilities of equating the limits on literature-reported validity coefficients to quantiles of the uncertainty distribution in the sensitivity analysis. From this sensitivity analysis result, the level of uncertainty assumed in the distribution of validity coefficient has negligible effect on the mean and the mode but not the median estimates of ${\widehat{\beta}}_{T_1}$ and ${\widehat{\beta}}_{T_2}$. As expected, the uncertainty in the estimates increases with the level of uncertainty assigned to the validity coefficients.

**Table 4:** The mean (standard deviation), median, 0.05 and 0.95 quantile and mode for logHR estimates for FV intake and for number of cigarettes smoked adjusted for the bias with the multivariate method, for various possibilities of equating the limits of literature-reported validity coefficients to quantiles of the uncertainty distribution, EPIC study 1992–2000

CI (%)	LogHR estimate for FV intake ${\widehat{\beta}}_{T_1}$				LogHR for cigarettes smoking ${\widehat{\beta}}_{T_2}$			
	mean (SD)	median	90 % CI	mode	mean (SD)	median	90 % CI	mode
80	-0.206 (0.155)	-0.156	-0.545, -0.072	-0.105	0.178 (0.128)	0.142	0.086, 0.381	0.142
90	-0.181 (0.090)	-0.157	-0.375, -0.078	-0.125	0.163 (0.079)	0.145	0.094, 0.294	0.125
95	-0.179 (0.080)	-0.158	-0.348, -0.088	-0.155	0.157 (0.056)	0.145	0.099, 0.257	0.122
99	-0.173 (0.065)	-0.160	-0.300, -0.095	-0.135	0.150 (0.035)	0.144	0.107, 0.215	0.131

*Abbreviation*: CI is level of uncertainty in the range of literature-reported validity coefficient ${\rho}_{T_i{Q}_i}$ expressed as a credible interval

## Discussion

In this study, we proposed a method that can be used to adjust for the bias in the diet-disease association caused by measurement error in reported dietary intake. Besides adjusting for the bias, the method can also adjust for confounding and measurement error in the confounder simultaneously. The strength of this method is that an investigator does not necessarily have to conduct a validation study, provided there is valid knowledge on the extent of measurement error in the self-report instruments that are used. Validation studies are usually very costly to conduct. Importantly, the method is very useful in conducting a sensitivity analysis to determine the threshold of measurement error and error correlation that leads to substantial change in the parameter estimate that quantifies the association of interest. We demonstrated how to combine external validation data with the observed data to adjust for the bias in the association. The method permits an investigator to either use prior information on the correlation between the errors in the dietary intake and the confounder measurements or on the correlation between their true intakes to estimate the covariance between true intakes. In the EPIC study example, the logHR estimate for FV intake adjusted for the bias with the multivariate method differed slightly from the estimate adjusted for the bias with the univariate method. The logHR estimates for cigarette smoking obtained with both bias-adjustment methods were similar. The similarity in the performance of the two methods in our example is due to weak negative error correlation assumed in this study, leading to minimal contamination effect of confounder measurement error. Sensitivity analysis, however, shows that the outcome of the two methods differs strongly when one assumes a strong error correlation. Further found through sensitivity analysis is that depending on the assumed magnitude of measurement error in cigarette smoking, the logHR estimate for FV intake can either be greater or smaller than the unadjusted estimate [5, 6, 14]. Notably, the error in cigarette smoking importantly affected the logHR estimate for FV intake, but not vice versa. This could be due to the stronger effect of cigarette smoking than FV intake on mortality and to the lesser measurement error assumed for cigarette smoking. In our method, we assumed there was no proportional scaling bias, as information on the magnitude of this bias was not available for FV intake and number of cigarettes smoked in a lifetime at the time of this study. However, the proposed method can be easily extended to incorporate such information. The same applies when an investigator wants to incorporate the effects of subject characteristics on their self-reports. In most cases there is no exact external information on the validity of self-report instruments. In such cases, the method allows the user to conduct a sensitivity analysis with a range of plausible estimates to explore the extent to which conclusions derived from the study could be influenced by measurement error. The method also allows pin-pointing assumptions that are crucial for drawing the right conclusion, so that future efforts can be directed towards obtaining valid information.

The main interest in this work was to demonstrate how to combine external validation data with the observed data and to explore the sensitivity of the adjusted estimates to the magnitude and correlation of measurement errors using a well-established multivariate method that applies attenuation-contamination matrix. We, nevertheless, conducted a simple simulation study to assess how well the multivariate method approximates true association parameters (see Additional file 1: Appendix D). From this simulation study, the multivariate method approximates log HR for FV intake more closely (bias = -0.004) than the univariate method (bias = -0.033) but with slightly larger uncertainty (std =0.085 vs std =0.082). In contrast, the unadjusted log HR estimate is severely biased (bias = 0.066) and with the smallest standard deviation as compared with those from the two adjustment methods.

This method, however, has a few limitations. First, we assumed an additive error structure for the DQ. Generally, however, some intake variables might exhibit multiplicative error structure, where the magnitude of measurement error increases with the quantity of intake [1, 38]. In a multiplicative error framework, a remedy could be transform the multiplicative error structure to an additive structure and then proceed with the proposed method. Second, the literature-reported data on validity coefficients for FV intake were based not on gold standards but on concentration markers and recall measurements that do not provide direct measures of true intake [39, 40]. Similarly, cotinine used as a marker for cigarette smoking suffers from the same limitation [34, 41]. Thus, the validity coefficients for these variables cannot be determined exactly [17, 34]. Nevertheless, the Bayesian MCMC sampling-based estimation approach used in the proposed method can still account for the uncertainties in the validity coefficients reported from the literature.

With our example, we illustrate two important features of exposure measurement error. First, measurement error in the confounder can cause bias in the diet-disease association even if dietary intake is measured exactly. Second, when several exposure variables are measured with correlated errors, it can be difficult to predict the direction and magnitude of the association between an exposure and outcome of interest.

## Conclusions

In conclusion, the proposed method can be used to adjust for the bias in the diet-disease association provided there is valid prior information on the magnitude of measurement error in the self-report instrument. The method allows the researcher to venture beyond general statements that measurement error in the confounders might have biased the results, because it allows an assessment of the sensitivity of the estimates to different assumptions regarding the structure of the measurement error. Our example illustrates the well-known fact that measurement error in a major risk factor (e.g., smoking) can affect the association estimate of a suspected risk factor (e.g., FV intake).

## Additional file

Additional file 1: Fisher-z transformation formula for generating validity coefficient, SAS macro for implementing the methods, simulation details and results using the methods shown in this work. (DOCX 222 kb)

## Abbreviations

DQ: Dietary questionnaire
EPIC: European prospective investigation into cancer and nutrition study
FV: Fruits and vegetables
LogHR: Logarithm of hazard ratio
MCMC: Markov Chain Monte Carlo
