# Supplementary material for: A method for sensitivity analysis to assess the effects of measurement error in multiple exposure variables using external validation data
Source: BMC Med Res Methodol. 2016 Oct 13;16:139. doi: 10.1186/s12874-016-0240-1 (PMC5064985; doi:10.1186/s12874-016-0240-1)
Supplement: Additional file 1: — Fisher-z transformation formula for generating validity coefficient, SAS macro for implementing the methods, simulation details and results using the methods shown in this work. (DOCX 222 kb) [file 12874_2016_240_MOESM1_ESM.docx]

**Additional file 1: “A method for sensitivity analysis to assess the effects of measurement error in multiple exposure variables using external validation data ”**

by

George O. Agogo, Hilko van der Voet, Pieter van’t Veer, Pietro Ferrari, David C Muller, Emilio Sánchez-Cantalejo, Christina Bamia, Tonje Braaten, Sven Knüppel, Ingegerd Johansson, Fred A. van Eeuwijk, and Hendriek Boshuizen

## Appendix A How to generate validity coefficients from the range of plausible values obtained from the literature review

Using Fisher z-transformation formula, the validity coefficient ($\rho_{T_{i}Q_{i}}$) for the *i*^th^ study variable is transformed as

${F_{z}}_{i}=\frac{1}{2} \ln\left( \frac{1+\rho_{T_{i}Q_{i}}}{1-\rho_{T_{i}Q_{i}}} \right)$, (1)

where ${F_{z}}_{i}'s$ are approximately normally distributed. We denote the lower and upper limits of the reported validity coefficients by $r_{1}$ and $r_{2}$ , respectively. We then use the ${F_{z}}_{i}$ formula (in expression 1) to obtain the corresponding Fisher z-transformed values for the upper and lower limits of the validity coefficient as ${F_{z}}_{1}$and ${F_{z}}_{2},$ respectively. Further, using the confidence interval formula for a standard normal random variable, we compute the mean $\mu_{Z_{i}}$and the variance $\sigma_{Z_{i}}$ of ${F_{z}}_{i}$ as $\mu_{z_{i}}=0.5({F_{z}}_{2}+{F_{z}}_{1} )$ and $\sigma_{z_{i}}=\frac{1}{2}\frac{({F_{z}}_{2}-{F_{z}}_{1})}{Z_{\alpha/2}}$, respectively, where $Z_{\alpha/2}$ is the $(1-\frac{\alpha}{2})$% quantile of a standard normal random variable. With this parameterization, the ${F_{z}}_{i}'s$ are generated as ${F_{z}}_{i}\sim N(\mu_{z_{i}}, \sigma_{z_{i}})$. Subsequently, the ${F_{z}}_{i}'s$ are transformed back to the validity coefficient using the inverse of Fisher z- transform as

$\rho_{T_{i}Q_{i}}=\frac{\exp\left( 2{F_{z}}_{i} \right)-1}{\exp\left( 2{F_{z}}_{i} \right)+1}$ . (2)

## Appendix B Distribution of correlation coefficients. Kernel densities and histograms for the distribution of validity coefficients for fruit and vegetable (FV) intake ($\rho_{Q_{1}T_{1}}$) and number of cigarettes smoked ($\rho_{Q_{2}T_{2}}$) as reported in the dietary questionnaires, generated from external validation data by assuming the reported lower and upper limits as 0.05 and 0.95 quantiles of the uncertainty distribution, respectively; the distribution of error correlation ($\rho_{\epsilon}$) was obtained based on the correlation in the observed data and prior information on the plausible sign of $\rho_{\epsilon}$ for FV intake and cigarette smoking as explained in the main text. Note, with the assumed quantile interval, it is possible to get a small positive value for the error correlation as shown in the distribution of $\rho_{\epsilon}$.

##
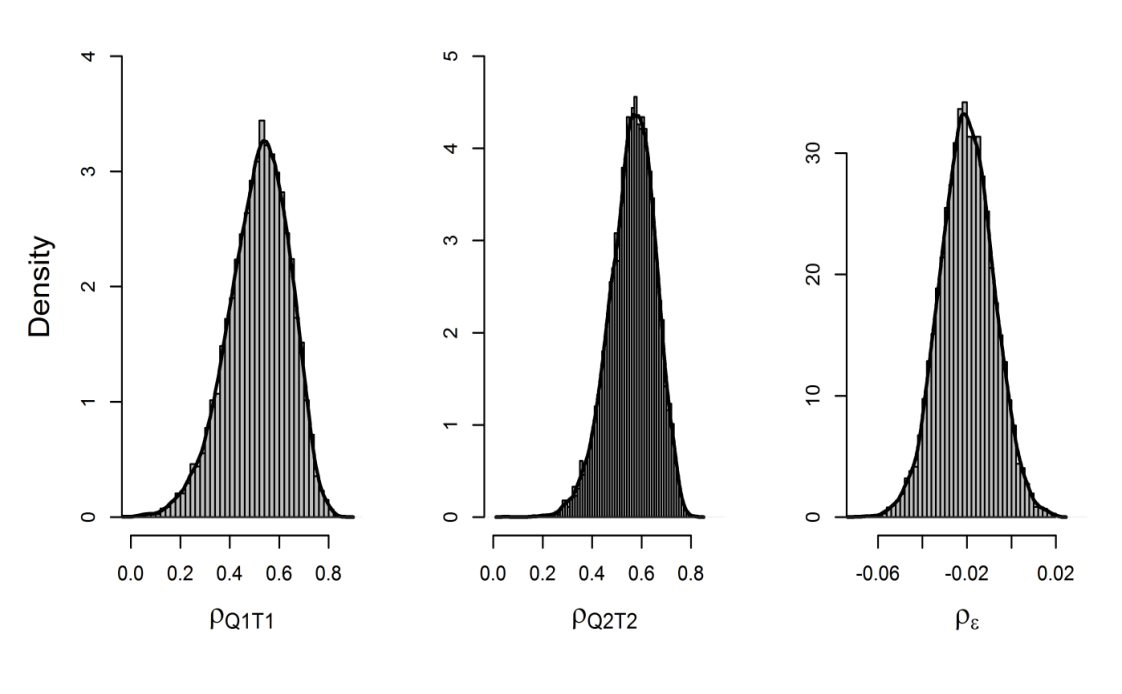


## Appendix C SAS macro implemented in version 9.3 for carrying out the proposed bias-adjustment method

/************* macro variable definitions****************************

****data: dataset containing the exposures measured with error

*****Q1=> self-report measurement for variable 1

*****Q2=> self-report measurement for variable 2

*r11-r12: lower & upper limits of validity coefficient for Q1: rho(T1,Q1)

**r21-r22: lower & upper limits of validity coefficient for Q2:rho(T2, Q2)

****ri's : validity coefficients of Qi in measuring Ti

****z_alpha: is the standard normal quantile for ri's

*rhot1-rhot2: lower & upper CI for correlation between true intakes t1&t2

***rhoe1-rhoe2: lower & upper CI for error correlation e1 & e2

****seed: random starting seed

****alpha11, alpha12=1: proportional scaling bias terms for Q1 and Q2

****rho_error: is the error correlation, rho (e1, e2)

****beta1: naive association estimate for variable 1, Q1

****beta2: naive association estimate for variable 2, Q2

****t_beta1-t_beta2: bias-adjusted estimates with multivariate method

***t_beta1_uni- t_beta2_uni:bias-adjusted estimates using univariate method

****pred: vector of covariates in the disease model

****nbi: number of burn in samples

****n=nmc=number of generated/mcmc samples

****thin: thinning rate

****ntu: number of samples used to tune the parameters

***********************/

**%macro** deatten(data=data,r11=**0.3**, r12=**0.7**, r21=**0.4** , r22= **0.7**, rhot1=, rhot2=, rhoe1=-**0.04**, rhoe2=**0**, r1=, r2=, alpha11= ,alpha12=,

z_alpha=**1.645**, seed=**284**, nmc=**50000**, nbi=**5000**, thin=**1**, ntu=**5000**);

%let nsim=&nmc/&thin; *number of MCMC samples use for the analysis;

%let q1=; * name of error prone var1 of interest in the dataset;

%let q2= ; * name of error prone var2 in the dataset;

%let pred= &q1 &q2; *measured covariates in the disease model;

%let time= ; * vent time;

%let death= ;

%let rho1= &rhot1 &rhoe1; * lower limit for either rhot1t2 or rhoe1e2;

%let rho2= &rhot2 &rhoe2; * upper limit for either rhot1t2 or rhoe1e2;

/************************Bayesian Cox analysis**************************/

proc sort data = data1; by descending &time; run;

data _null_;

set data1 nobs=_n;

call symputx('N', _n); stop; run;

proc freq data=data1;

ods select none;

tables &time / out=freqs; run;

proc sort data = freqs; by descending &time; run;

data data1; set data1;ind = _N_; run;

ods select all;

ods graphics on;

proc mcmc data=data1 outpost=deattenuated nmc=&nmc nbi=&nbi seed=&seed PLOTS=(TRACE AUTOCORR) thin=&thin ntu=&ntu;

array beta[**2**];

parms beta: **0** z1 **0** z2 **0** z3 **0**;

prior beta: ~ normal(**0**, var=**1e6**);

bZ = beta1 * &q1 + beta2 * &q2;

if ind = **1** then do; /* first observation */

S = exp(bZ);

l = &death * bZ;

v = &death;

end;

else if (**1** < ind < &N) then do;

if (lag1(&time) ne &time) then do;

l = &death * bZ;

l = l - v * log(S); /* correct the loglike value */

v = &death; /* reset v count value */

S = S + exp(bZ);

end;

else do; /* still a tie */

l = &death * bZ;

S = S + exp(bZ);

v = v + &death; /* add # of noncensored values */

end;

end;

else do; /* last observation */

if (lag1(&time) ne &time) then do;

l = - v * log(S); /* correct the loglike value */

S = S + exp(bZ);

l = l + &death * (bZ - log(S));

end;

else do;

S = S + exp(bZ);

l = &death * bZ - (v + &death) * log(S);

end;

end;

model general(l);

/****************posterior sample for covariance of Q************/

array data[**2**] &pred;

array mu[**2**];

array Sigma[**2**,**2**];

array mu0[**2**] (**0** **0**);

array Sigma0[**2**,**2**] (**1e6** **0** **0** **1e6**);/******vague prior for the mean********/

array Scale[**2**,**2**] (**1** **0** **0** **1**);

parm mu Sigma;

prior mu ~ mvn(mu0, Sigma0);

prior Sigma ~ iwish(**2**, Scale);/***********LI iWishart***************/

model data ~ mvn(mu, Sigma);

/****************Fisher Z values for validity coefficients: rho T,Q ***/

z11=**0.5***log((**1**+&r11)/(**1**-&r11));*LL z1;

z12=**0.5***log((**1**+&r12)/(**1**-&r12));*UL z1;

* var2;

z21=**0.5***log((**1**+&r21)/(**1**-&r21)); *LL z2;

z22=**0.5***log((**1**+&r22)/(**1**-&r22)); *UL z2;

* means of Fishers Z-transform from CI formula;

mu_z1=**0.5***(z12+z11); *mean z1;

mu_z2=**0.5***(z22+z21); *mean z2;

*Standard errors from CI formula;

se1=(z12-z11)/(**2***&z_alpha);

se2=(z22-z21)/(**2***&z_alpha);

*generating a vector of Fisher Z random values from external data;

prior z1 ~ normal(mu_z1, var = se1*se1);

prior z2 ~ normal(mu_z2, var = se2*se2);

*****rho t1t2 or rho e1e2 whichever information is available;

z31=**0.5***log((**1**+&rho1)/(**1**-&rho1));*LL z1;

z32=**0.5***log((**1**+&rho2)/(**1**-&rho2));*UL z1;

mu_z3=**0.5***(z32+z31); *mean rhot1t2;

se3=(z32-z31)/(**2***&z_alpha);

prior z3 ~ normal(mu_z3, var = se3*se3);

run; ods graphics off;quit;

* correlation coefficients using inverse Fisher-z transform formula;

data deattenuated; set deattenuated;

r1=(exp(**2***z1)-**1**)/(exp(**2***z1)+**1**);

r2=(exp(**2***z2)-**1**)/(exp(**2***z2)+**1**);

rename Sigma1=varq1 sigma2=covq1q2 sigma4=varq2; run;

data deattenuated ; set deattenuated;

sigq1=sqrt(varq1); sigq2=sqrt(varq2);

corr_q1q2=covq1q2/(sigq1*sigq2); * correlation between Q1 and Q2;

* std for true values using validity coefficient rhoT,Q formula;

t1_sig=(r1*sigq1)/&alpha11;

t2_sig=(r2*sigq2)/&alpha12;

* variances for t1 & t2;

t1_var=t1_sig****2**;

t2_var=t2_sig****2**;

*std of measurement error;

sig_err1= sqrt(varq1-t1_var);

sig_err2= sqrt(varq2-t2_var);

/*****correlation from either rhoT1T2 or rhoe1e2**************************/

r3=(exp(**2***z3)-**1**)/(exp(**2***z3)+**1**);

/***** calculating covT1T2 variable from either rhoT1T2 or rhoe1e2*/

if %sysevalf(&rhot1^= AND &rhot2^= ) then

covt1t2=r3*t1_sig*t2_sig; *covt1t2 when rhoT1T2 is available;

else

covt1t2= covq1q2-(r3*sig_err1*sig_err2);*covt1t2 when rhoe1e2 is available;

/******************calculating rhoT1T2 and rhoe1e2*********************/

rho_t1t2=covt1t2/(t1_sig*t2_sig);

rho_error=(covq1q2-covt1t2)/(sig_err1*sig_err2);

/**************inverse of attenuation-contamination***** matrix*****/

k=(varq1*varq2)-(covq1q2****2**); *determinant of Q matrix;

*elements of inverse of Q matrix;

x1=varq2/k ; x2=((-**1**)*covq1q2)/k; x3=x2; x4=varq1/k ;

*elements of attenuation matrix {a1 a2, a3 a4};

a1=(x1*t1_var)+(x3*covt1t2);

a2=(x2*t1_var)+(x4*covt1t2);

a3=(x1*covt1t2)+(x3*t2_var);

a4=(x2*covt1t2)+(x4*t2_var);

* elements of inverse of the transposed attenuation matrix {i1 i2, i3 i4};

det=(a1*a4)-(a2*a3); *determinant;

i1=a4/det; i2=((-**1**)*a3)/det; i3=((-**1**)*a2)/det; i4=a1/ det;

* computing the de-attenuated coefficients;

t_beta1=(i1*beta1)+(i2*beta2);

t_beta2=(i3*beta1)+(i4*beta2);

*univariate de-attenuation;

t_beta1_uni=(varq1/t1_var)*beta1; t_beta2_uni=(varq2/t2_var)*beta2;

drop k x1 x2 x3 x4 a1 a2 a3 det LogPrior LogLike mu1 mu2 k

LogPost a1 a2 a3 a4 det x1 x2 x3 x4 sigma3 Iteration;run;

ods select all;

/***********outputting summary statistics********************************/

proc means data=deattenuated mean median std p5 p95 ndec=**3** ;

var t_beta1 t_beta2 t_beta1_uni t_beta2_uni beta1 beta2 rho_t1t2 r1 r2 covt1t2 rho_error ;title 'Summary measures'; run;

/*univariate kernel smoothing for the de-attenuated betas*****************/

proc kde data=deattenuated; univar t_beta1/ bwm=**2** out=k_beta1 ;run;

proc kde data=deattenuated; univar t_beta2/ bwm=**2** out=k_beta2 ;run;

/*************bias-adjusted beta with maximum kernel density***************

***************i.e., the mode of de-attenuated****************************/

proc sort data=k_beta1; by density ; run;

proc sort data=k_beta2; by density ; run;

/************************mode beta1**************************************/

proc sql noprint; select n(density) into: nobs from k_beta1;quit;

proc print data=k_beta1(firstobs=&nobs); title 'mode t_beta1'; run;

/*************************mode beta2************************************/

proc sql noprint; select n(density) into: nobs1 from k_beta2;quit;

proc print data=k_beta2(firstobs=&nobs1); title 't_mode beta2' ;run;

**%mend**;

%***deatten***();

**Appendix D** Simulation study set up

We conducted a simple simulation to assess the performance of the proposed multivariate method for adjusting for bias in the association parameter for two variables measured with correlated errors. The following steps were followed.

1. We generated the true distributions of the validity coefficients ($\rho_{Q_{i}T_{i}}$) for FV intake (limits: 0.3 to 0.7) and cigarettes smoking (limits: 0.4 to 0.7), and correlation coefficient ($\rho_{T_{1}T_{2}}$) between their true intakes (limits: -0.3 to -0.1) using Fisher Z-transform formula. These ranges were assumed as 90% CI.
2. We generated correlated true intake values for FV intake ($T_{1}$, per 100g/day) and cigarette smoking ($T_{2}$) following a normal distribution as follows

$T_{1}=\mu_{T_{1}}+\epsilon_{T_{1}}\times\sigma_{T_{1}};\epsilon_{T_{1}}\sim N(0,1)$

$T_{2}=\mu_{T_{2}}+\xi_{T_{2}}$,

where $\xi_{T_{2}}=\rho_{T_{1}T_{2}}\times\sigma_{T_{2}}\times\epsilon_{T_{1}}+\sigma_{T_{2}}\times\epsilon_{T_{2}}\sqrt{(1-\rho_{T_{1}T_{2}}^{2})}; \epsilon_{T_{2}}\sim N(0,1)$ i.e. using Cholesky decomposition.

1. We generated measurement error variance as

$$\sigma_{\epsilon_{Q_{i}}}^{2}=\sigma_{T_{i}}^{2}\left( \frac{1-\rho_{T_{1}T_{2}}^{2}}{\rho_{T_{1}T_{2}}^{2}} \right)$$

2. The intakes measured with error were generated following a classical measurement error as follows

$$Q_{i}=T_{i}+\epsilon_{Q_{i}};\epsilon_{Q_{i}}\sim N\left( 0,\sigma_{\epsilon_{Q_{i}}} \right)$$

3. The exponentially distributed time to death was generated dependent on FV intake and cigarette smoking. Further, individuals with time to death greater than the 25th percentile of the distribution of time to death were censored. Survival time was defined as the minimum of the two times, i.e., time to death and censoring time. In generating time to death, the log hazard ratio for FV intake was taken as -0.1 and for cigarette smoking as 0.1.
4. We applied the multivariate bias-adjustment method to the simulated dataset and compared the results with those from the univariate adjustment method and the naïve method that ignores measurement error. The analysis was based on 50 000 posterior samples, after discarding 5000 burn-in samples and using 5000 samples to tune the parameters. The results were summarized using mean, median and standard deviation of logHR estimates.

**Simulation results**

The simulation results are presented in Table D. The multivariate method approximates log HR for FV intake more closely (bias =-0.004) than the univariate method (bias=-0.033) but with slightly larger uncertainty (std=0.085 vs std=0.082). In contrast, the unadjusted log HR estimate is severely biased (bias=0.066) and with the smallest standard deviation. The small standard deviation for the unadjusted log HR is because there is no extra uncertainty attributable to calibration. A similar trend is observed in the log HR estimates for cigarette smoking.

Table A1 The mean, median and standard deviation for the Log Hazard Ratio (logHR) estimates for FV intake (per 100gram per day) and average number of cigarettes smoked (per day) adjusted for the bias with multivariate and univariate methods, and also the unadjusted estimates that ignores measurement error in a simulation study

|  | LogHR for FV intake $\hat{\beta}_{T_{1}}$ | | | |  | LogHR for cigarettes smoking $\hat{\beta}_{T_{2}}$ | | | |
| --- | --- | --- | --- | --- | --- | --- | --- | --- | --- |
| methods | | mean | bias | median | std | mean | bias | median | std |
| True value | | -0.100 | 0.00 | -0.100 | 0.030 | 0.100 | 0.00 | 0.100 | 0.030 |
| Multivariate | | -0.104 | -0.004 | -0.092 | 0.085 | 0.095 | -0.005 | 0.091 | 0.065 |
| Univariate | | -0.133 | -0.033 | -0.121 | 0.082 | 0.118 | 0.008 | 0.112 | 0.062 |
| Unadjusted | | -0.034 | 0.066 | -0.034 | 0.015 | 0.036 | -0.064 | 0.036 | 0.017 |
